# Multi-Omics and Liquid Biopsy Integration for Precision Stratification of Breast Cancer

**DOI:** 10.3390/cancers18193235

**Published:** 2026-10-08

**Authors:** Dheeraj Sharma, Shriyansh Srivastava, Sathvik Belagodu Sridhar, Javedh Shareef, Rakesh Sahu, Roja Rani Budha, Mohd Tariq, Mohammad Amjad Kamal

**Affiliations:** 1 Department of Pharmacology, College of Pharmacy, JSS University, Noida 201301, Uttar Pradesh, India; 2 Department of Pharmacy, School of Medical and Allied Sciences, Galgotias University, Greater Noida 203201, India; 3 College of Pharmacy, Dubai Medical University, Dubai P.O. Box 20170, United Arab Emirates; 4 RAK College of Pharmacy, RAK Medical & Health Sciences University, Ras al Khaimah P.O. Box 11172, United Arab Emirates; 5 School of Pharmacy, Sharda University, Knowledge Park III, Greater Noida 201310, India; rakeshsahu1100@gmail.com; 6 School of Pharmacy, D Y Patil Deemed to be University, Sector 7, Nerul, Navi Mumbai 400706, India; 7 Department of Biotechnology, Graphic Era (Deemed to be University), Dehradun 248002, Uttarakhand, India; 8 Future Technologies Research Centre, King Faisal University, P.O. Box 400, Al Ahsa 31982, Saudi Arabia; 9 Novel Global Community Educational Foundation, Hebersham, NSW 2770, Australia

**Keywords:** breast cancer diagnostics, circulating tumor DNA (ctDNA), liquid biopsy, molecular signatures, precision oncology, gene expression profiling

## Abstract

Breast cancer is a biologically heterogeneous disease that requires accurate diagnosis, risk stratification, treatment selection and longitudinal assessment. Conventional imaging and tissue biopsy remain the mainstays of clinical management. However, tissue sampling represents a specific anatomical site and time point, and may not reflect the molecular heterogeneity or evolution of disease. Liquid biopsy offers a minimally invasive complementary approach, analyzing circulating tumor cells (CTCs), circulating tumor DNA (ctDNA) and extracellular vesicles. Applications include molecular characterization, treatment-response assessment, resistance detection and minimal residual disease (MRD) evaluation, which are under investigation or in clinical use. Gene-expression profiles, mutational alterations and epigenetic marks can generate molecular signatures that offer further support for tumor classification and risk stratification, and have demonstrated clinical value in specific breast cancer populations. Advances in next-generation sequencing, digital PCR, proteomic analysis, and artificial intelligence have expanded the analytical capacity for detection and interpretation of circulating and tissue-derived biomarkers. However, biological heterogeneity, low abundance of circulating biomarkers, pre-analytical and analytical variability, standardization of assays and limited prospective evidence for some applications are still major barriers to clinical translation. In this review, we discuss the biological basis, detection technologies, clinical applications and limitations of liquid biopsies and molecular signatures in breast cancer, highlighting multi-omics integration and computational approaches. It distinguishes between promising analytical biomarkers and clinically validated applications and highlights the evidence and methodological needs for wider implementation in precision breast cancer care.

## Simple Summary

Breast cancer is a heterogenous disease, and conventional imaging and tissue biopsy may not always reflect the changes occurring throughout the tumor over time. Liquid biopsy is a less invasive approach to study tumor-related material in blood, and molecular signatures can give information on tumor biology, disease risk and response to treatment. This review covers circulating tumor cells, circulating DNA, extracellular vesicles, gene-expression patterns, mutational signatures and microRNAs, and the technologies used to detect them. It discusses their current clinical applications, limitations and emerging roles in personalized breast cancer care. This review integrates evidence from liquid biopsies, molecular diagnostics, multi-omics approaches, and artificial intelligence to point out knowledge gaps and areas needing further validation. These findings may serve to guide future research for reliable and clinically useful molecular diagnostic strategies.

## 1. Introduction

Breast cancer is a heterogeneous malignancy with histopathological features, receptor status, genomic alterations and patterns of tumor evolution that all contribute to diagnosis, prognosis and therapeutic response. Early and accurate detection is clinically important, because disease detected at an earlier stage generally has a better prognosis [1]. The current diagnosis is therefore based on complementary methods such as breast imaging, histopathological examination, immunohistochemistry and molecular testing. Mammography is still the gold standard for breast cancer screening, but breast density can affect the diagnostic performance of mammography, leading to missed or indeterminate findings [2]. Ultrasound and magnetic resonance imaging (MRI) add further anatomical information but usually need to be correlated with clinical, pathological or molecular findings, rather than being stand-alone methods for definitive tumor characterization [3]. A tissue biopsy is still required to confirm malignancy and to define pathological and receptor-defined characteristics including estrogen receptor (ER), progesterone receptor (PR), human epidermal growth factor receptor 2 (HER2) and proliferation-associated markers such as Ki-67 [4].

Although important for diagnosis, a tissue sample only provides information about a small area of a tumor at a specific time point. In particular, this is relevant to breast cancer where genetically and phenotypically distinct subclones may be present in the primary tumor and may differ from lesions arising during metastatic progression or after treatment. Therapeutic pressure may also further alter the clonal composition of the disease by selecting for resistant populations, leading to temporal changes in molecular characteristics that may not be detected in a single biopsy [1,5]. Repeated tissue sampling may therefore be difficult to perform and may not reflect the evolving systemic disease adequately. These limitations have generated interest in methods that can complement tissue-based assessment with molecular information obtained longitudinally.

One such approach is liquid biopsy, which is the analysis of tumor-associated material in accessible body fluids, most notably blood. Circulating tumor cells (CTCs), cell-free DNA (cfDNA), circulating tumor DNA (ctDNA) and extracellular vesicles carrying nucleic acids and proteins may be released by tumor-cell death, cellular shedding or active secretion [6]. We can get molecular information from their analysis without having to repeatedly collect tissue invasively. However, the biological contribution of each analyte depends on tumor burden, vascular access, cellular phenotype, shedding characteristics and disease stage; thus, a circulating signal should not be considered an exact representation of every tumor compartment [7]. This limitation is particularly pertinent in early-stage disease where the amount of tumor derived material may be limited, and normal circulating material can provide a substantial analytical background. Thus, liquid biopsy is currently used as a complementary molecular approach, not as a universal replacement for tissue diagnosis [8].

Molecular signatures together with liquid biopsy offer a framework for describing the biological state of breast tumors beyond the traditional clinicopathological classification [9]. Gene-expression profiles can capture coordinated transcriptional programs associated with proliferation and hormone signaling, while mutational and epigenetic signatures can inform about genomic instability, tumor evolution and potentially actionable molecular alterations [9,10]. Molecular risk stratification has shown its value in selected breast cancer populations using clinically established gene-expression assays. Circulating biomarkers are increasingly being studied for treatment-response assessment, resistance detection, and minimal residual disease (MRD) evaluation [11]. Critically, the clinical relevance of these approaches is dependent on the biomarker, disease setting, assay and level of prospective validation; analytical detection alone does not establish clinical utility or demonstrate that action on a molecular result improves patient outcomes [12].

The convergence of liquid biopsy, molecular profiling, advanced analytical technologies, and computational approaches is an evolving component of precision breast cancer diagnostics [13]. This review discusses the biological basis of liquid biopsy biomarkers and molecular signatures such as CTCs, ctDNA, extracellular vesicles, gene-expression patterns, mutational signatures and microRNAs. It further deliberates on the technologies for their detection, current and emerging clinical applications, commercially available molecular assays and the roles of artificial intelligence and multi-omics integration. Special attention is given to biological and technical factors affecting biomarker interpretation, such as tumor heterogeneity, low circulating biomarker abundance, and assay standardization and reproducibility. The objective of this review is to outline the state of the art of diagnostic value and remaining translational challenges for liquid biopsies and molecular signatures in breast cancer, separating clinical established and investigational applications.

## 2. Liquid Biopsies in Breast Cancer

Tissue biopsy plays a central role in breast cancer diagnosis, but a single specimen is representative of a particular tumor site and time point and may not reflect the heterogeneity of evolving disease [14,15,16]. Liquid biopsy offers a minimally invasive complementary approach to characterize tumor-associated material in biofluids, especially blood, such as circulating tumor cells (CTCs), circulating tumor DNA (ctDNA) and extracellular vesicles [15,16,17,18,19]. Serial liquid-biopsy sampling may offer longitudinal molecular information during disease progression and treatment [20,21,22]. However, circulating biomarkers are not representative of all tumor sites and applications such as molecular residual disease assessment and resistance detection need proper clinical validation [17,23,24,25]. Liquid biopsy, therefore, should be considered as an adjunct to tissue-based analysis, rather than as a panacea.

### 2.1. Principles and Advantages of Liquid Biopsies

Liquid biopsy is based on the detection of tumor-associated biological material shed into accessible body fluids, most commonly peripheral blood [26]. Tumor-derived material enters the circulation through a number of biological processes including apoptosis and necrosis that release fragmented nucleic acids and other intracellular components, as well as active cellular secretion and extracellular-vesicle-mediated transport [27,28]. The circulating generated material consists primarily of molecules derived from normal tissues, while the tumor-derived fraction depends on tumor burden, vascular access, disease stage, cellular phenotype, and biological activity [20]. Therefore, the diagnostic principle of liquid biopsy relies not only on the detection of circulating nucleic acids or cells but also on the discrimination of tumor-associated signals from a significantly larger background of non-tumor material [29]. Analytical platforms like digital PCR and next-generation sequencing (NGS) improve the ability to detect low-frequency molecular alterations, but their performance is still conditioned by sample quality, target abundance, assay design and analytical depth [30,31].

One of the main advantages of liquid biopsy is the possibility of serial sampling with relatively low procedural burden. Tissue biopsy provides molecular information from a specific anatomical site at a particular time point, whereas repeated blood sampling can be performed during therapy to track changes in circulating biomarkers over time [32]. This allows for longitudinal monitoring of molecular evolution, such as the emergence or expansion of treatment-resistant clones, without the need for repeated invasive tissue collection [33]. However, a circulating biomarker is a measure of the material that is released into the compartment being sampled and not a census of all sites of tumors. Thus, absence of a detectable signal does not necessarily imply absence of disease. This distinction is especially relevant in metastatic disease and low-burden disease, where differences in tumor vascularization, shedding rate and metastatic site can impact the detectability of biomarkers [34].

Liquid biopsy also provides an important paradigm for the study of minimal residual disease (MRD) and molecular recurrence. Residual tumor cells after apparently successful treatment may be present below the detection limit of conventional imaging but continue to shed small quantities of tumor-associated DNA or other biomarkers [35]. Therefore, sensitive molecular assays may detect persistent or re-emergent tumor-derived signals in some patients prior to radiological evidence of progression [36]. However, the detection of a circulating signal is evidence of an increased likelihood of residual disease or recurrence, but should not be automatically interpreted as evidence that changing therapy based on that result improves clinical outcome [36,37]. Similarly, although in some cases molecular alterations from the tumor can be detected before radiological evidence of a lesion, liquid biopsy as a population-wide screening or definitive early-detection test remains under active clinical investigation [38,39].

Therefore, the main value of liquid biopsy is its minimally invasive, repeatable access to molecular information that complements but does not replace tissue-based diagnosis universally [40]. Its applications extend from molecular characterization and longitudinal monitoring of disease to the investigation of treatment resistance and MRD [25]. The power of the approach is based on the biological properties of the analyte and on the analytical performance of the assay, while low tumor-derived abundance, biological heterogeneity, preanalytical variation and the need for clinical validation are still important limitations [30]. These considerations are the basis for the consideration of the individual biomarker classes, CTCs, ctDNA and extracellular vesicles, and their distinct biological and diagnostic properties in the following sections [19].

### 2.2. Types of Biomarkers in Liquid Biopsies

**Figure 1:**
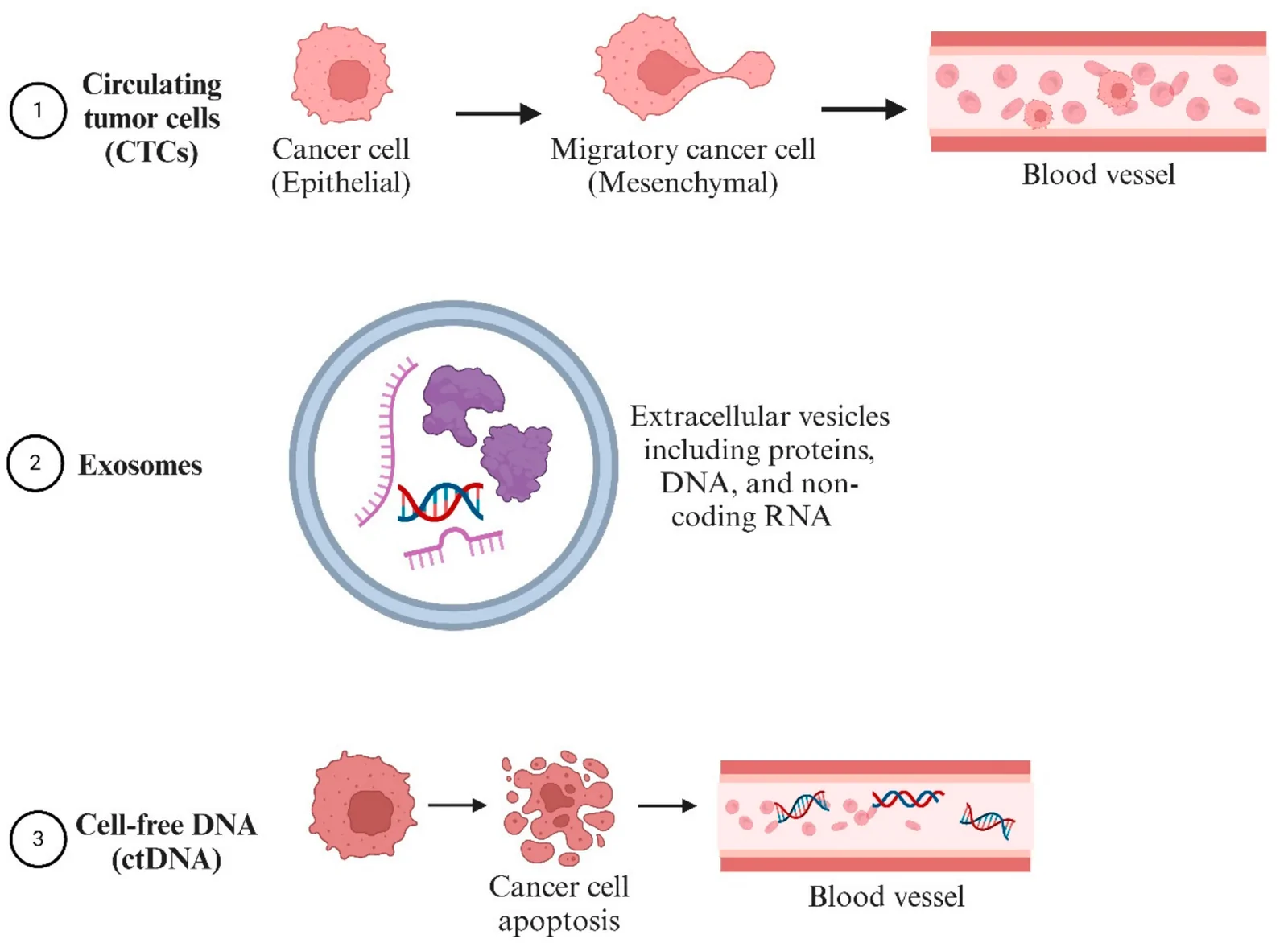
Types of biomarkers in liquid biopsies for breast cancer diagnosis.

A liquid biopsy can define several different tumor-derived entities, each of them providing a specific kind of biological information. The three major classes of biomarkers used in cancer detection, prognosis, and therapeutic decision-making are circulating tumor cells (CTCs), circulating tumor DNA (ctDNA), and exosomal RNA [24], Figure 1.

#### 2.2.1. Circulating Tumor Cells

Circulating tumor cells (CTCs) are intact tumor cells that are shed from primary or metastatic lesions into the bloodstream [16,19,41]. Compared to cell-free nucleic acids, CTCs preserve cellular morphology and can provide information on tumor-cell phenotype, viability and heterogeneity [15,17,42,43,44,45]. However, their abundance is usually very low and dependent on tumor burden, disease stage, metastatic site and tumor-cell shedding, requiring specialized enrichment and detection methods [17,25,46,47].

CTCs can be isolated by immunoaffinity, size-based, density-based or microfluidic approaches and identified by epithelial, mesenchymal or tumor associated markers [17,24,25]. Different platforms can capture different CTC populations and produce non-interchangeable results as epithelial-to-mesenchymal transition can downregulate the expression of epithelial-marker [15,24,48].

Besides enumeration, CTCs can be molecularly characterized for receptor expression, genomic alterations, and other features that are associated with metastatic potential or treatment resistance [15,21,49]. In breast cancer, higher CTC counts are generally correlated with poorer outcomes, especially in the metastatic setting [16,17]. However, CTC measurements are still affected by biological shedding and analytical platform bias [17,24]. The prognostic value of CTCs needs to be differentiated from the evidence that CTC-guided therapy improves patient outcomes [21,50]. Therefore, CTC analysis currently provides complementary prognostic and biological information, while broader treatment-guiding applications will require standardized assays and prospective validation [15,29,30,32].

#### 2.2.2. Cell-Free DNA

Cell-free DNA (cfDNA) consists of extracellular DNA fragments released into circulation from normal and malignant cells. Circulating tumor DNA (ctDNA) is the tumor-derived fraction of cfDNA [51]. Therefore, ctDNA can contain tumor-specific sequence alterations, copy-number changes, and other molecular features that provide insight into the genetic profile of the malignancy [52]. It is mainly released during apoptosis and necrosis of tumor cells, but active cellular processes and extracellular-vesicle-associated pathways may also play a role. The abundance of ctDNA in total cfDNA is highly variable and dependent on tumor burden, disease stage, anatomical site, vascularization and biological properties of individual tumor clones. Therefore, detection can be limited due to low ctDNA abundance, particularly in low volume disease [42,51,53].

The diagnostic value of ctDNA is based on the detection of low-frequency tumor-associated alterations by highly sensitive techniques such as digital PCR and targeted or deep NGS [42]. Serial ctDNA analysis can provide a molecular snapshot, but also show changes in tumor clones in the course of treatment [54]. Acquired endocrine resistance in hormone receptor-positive, HER2-negative breast cancer is a clinically relevant example, where mutations such as ESR1-Y537S and ESR1-D538G alter the ligand binding domain of estrogen receptor-α (ERα) [55]. These changes lock the receptor in an active shape, so that it can conduct ER-dependent transcription and tumor-cell proliferation even in the absence of estrogen, due to aromatase inhibitors. Therefore, detection of emerging ESR1 mutations in plasma ctDNA can identify a molecular mechanism of acquired endocrine resistance and inform consideration of an alternative endocrine strategy, including an estrogen receptor degrader, rather than continuing with an aromatase inhibitor alone [55,56].

#### 2.2.3. Exosomes

Extracellular vesicles (EVs), such as exosomes, are particles enclosed in a membrane and secreted by cells, containing proteins, lipids, DNA, mRNA and non-coding RNAs including microRNAs (miRNAs) [57]. EVs derived from tumors can enter the circulation and mediate intercellular communication by delivering their molecular cargo to recipient cells. Such transfer can modulate signaling pathways in tumor cells, as well as the surrounding microenvironment, in breast cancer, including pathways that control proliferation, invasion, immune regulation and treatment resistance [58]. Importantly, the lipid bilayer surrounding the EV cargo affords partial protection from extracellular RNases, thereby increasing the stability of vesicle-associated nucleic acids in blood relative to many freely circulating RNA molecules and supporting their investigation as liquid-biopsy biomarkers [59]. More broadly, metabolic reprogramming and alterations in the tumor microenvironment more generally may contribute to breast cancer development and to the molecular features of circulating tumor-associated material, highlighting that extracellular-vesicle signals should not be assumed to be exclusively tumor-derived [60,61].

Exosomal miRNAs are of special interest because their abundance and composition can be representative of tumor-associated molecular programs. In breast cancer, several studies have reported patterns of exosomal RNA associated with subtypes [62]. For example, miR-223 and miR-375 have been studied in the setting of luminal breast cancer while miR-21 and miR-155 are often detected among exosomal miRNAs associated with more proliferative or aggressive disease phenotypes [63]. Mechanistically, miR-21 can target tumor suppressive pathways such as PTEN signaling while miR-155 can target regulators of inflammatory and survival pathways such as SOCS1 [64]. However, these associations should be regarded as candidate molecular signatures rather than definitive subtype classifiers, as exosomal RNA profiles may differ depending on the tumor burden, cellular source, disease stage and the methods used for EV isolation and RNA measurement [65]. 

The main diagnostic potential of exosomes is to get relatively stable, biologically informative molecular material from blood, which can be repeatedly sampled [66]. However, for clinical translation, there is a need for standardized approaches to EV isolation, characterization, normalization, and cargo analysis, and prospective validation to demonstrate that an exosomal biomarker provides information beyond established clinicopathological variables [65] Table 1.

## 3. Molecular Signatures in Breast Cancer

Breast cancer is a biologically heterogeneous disease with distinct genomic, transcriptomic, epigenetic and phenotypic features. Histopathology and expression of estrogen receptor (ER), progesterone receptor (PR) and human epidermal growth factor receptor 2 (HER2) are still fundamental for diagnosis and treatment selection and form the basis of traditional classification [72]. Yet, molecular profiling has revealed considerable biological heterogeneity within and between these clinicopathological groups. Molecular signatures reflect coordinated patterns of gene expression, genomic alterations and non-coding RNA regulation that can inform tumor biology, recurrence risk and, in selected clinical settings, the potential benefit of systemic therapy [73]. High-throughput techniques such as RNA sequencing, next-generation sequencing (NGS) and whole-exome sequencing have allowed the characterization of these molecular features and have contributed to the development of clinically validated multigene assays [74].

### 3.1. Gene Expression Profiling

Gene expression profiling (GEP) characterizes the transcriptional activity of multiple genes simultaneously, providing a broader representation of tumor biology than immunohistochemistry (IHC) assessment of a small number of protein markers [75]. Seminal expression-profiling studies identified intrinsic breast cancer subtypes with distinct transcriptional programs, including Luminal A, Luminal B, HER2-enriched and basal-like tumors [76]. The original classification also described a normal-like group. These intrinsic subtypes are characterized by differences in hormone-receptor signaling, proliferation, HER2-related signaling, and other biological pathways and are associated with differences in clinical behavior and therapeutic sensitivity [77]. Luminal tumors are generally enriched for transcriptional programs associated with estrogen receptors, while basal-like tumors are often characterized by high proliferation and lack expression of ER, PR and HER2 at the conventional clinicopathological level [78]. Importantly, molecular intrinsic subtype and IHC-defined receptor status are not fully interchangeable, and molecular assays should be interpreted within the broader clinical and pathological context [78].

The clinical translation of GEP has led to several multigene assays directed at various clinical questions. Oncotype DX provides prognostic information and helps decide about adjuvant chemotherapy in selected patients with early-stage hormone-receptor-positive, HER2-negative breast cancer by measuring the expression of 21 genes to generate a recurrence score [79]. MammaPrint assesses a 70-gene expression signature to categorize patients based on genomic recurrence risk, while Prosigna employs the PAM50 gene-expression framework to determine intrinsic subtype and generate a risk-of-recurrence score [80,81]. These assays should not be considered interchangeable because they are based on different gene sets, analytical algorithms, patient populations and clinical end points. Hence, their clinical utility is dependent on the specific assay and clinical context, and not on gene-expression profiling as a uniform methodology.

At the biological level, gene-expression signatures result from coordinated changes in transcription factor activity, signal transduction pathways, chromatin state and non-coding RNA-mediated regulation [82]. Transcriptional factors such as FOXA1 and GATA3 are involved in the maintenance of luminal transcriptional programs, while alterations involving regulators such as TP53 may alter cell-cycle control, genomic stability, and downstream gene-expression patterns [83]. Epigenetic mechanisms like DNA methylation, histone modifications and chromatin remodeling further influence transcriptional states and may explain variation between tumors and between subclones of the same tumor [14]. These mechanisms may help explain why expression signatures can capture biological information not fully captured by individual receptor measurements. Adding transcriptomic data to genomic and epigenomic data may provide additional biological resolution, but the analytical and clinical problems of multi-omics integration are discussed separately in Section 9.1.

### 3.2. Mutational Signatures

Mutational signatures are distinct patterns of DNA sequence changes caused by particular endogenous or exogenous mutagenic processes. Their analysis can provide information about the mechanisms responsible for genomic damage, including defects in DNA repair, and can thus complement the identification of individual driver mutations [84]. Whole-genome and whole-exome sequencing have facilitated the characterization of recurrent mutational processes in breast cancer, uncovering considerable diversity in the mechanisms shaping tumor genomes [85]. Alteration of common cancer-associated genes such as TP53, PIK3CA, GATA3, BRCA1/2 and ESR1 are observed but their frequency and biological significance varies across breast cancer subtypes [86]. Crucially, these individual genomic alterations should be distinguished from mutational signatures: a PIK3CA mutation is a specific genetic alteration, while a mutational signature represents a broader pattern generated by an underlying mutagenic process [87].

PIK3CA is a gene that is often mutated in hormone receptor-positive breast cancer. The mutation can activate the phosphatidylinositol 3-kinase (PI3K)-AKT-mTOR pathway, which promotes cell survival, growth and proliferation [88]. Identifying activating PIK3CA alterations can have therapeutic relevance as PI3Kα inhibition with agents such as alpelisib has been evaluated in appropriately selected patients with PIK3CA-mutated, hormone receptor-positive, HER2-negative advanced breast cancer [89]. Similarly, acquired ESR1 mutations may affect estrogen-receptor signaling and contribute to endocrine resistance, and their detection is relevant to molecular monitoring and treatment selection. These are examples of clinically relevant genomic alterations, not mutational signatures [90].

A major group of mutational phenotypes in breast cancer is linked with defective DNA-damage repair. Homologous recombination repair is important for accurate DNA double-strand break repair and maintenance of genomic integrity and is dependent on BRCA1 and BRCA2 [85]. Loss of BRCA1/2 function can lead to homologous recombination deficiency (HRD), which results in genomic instability and reliance on alternative DNA-repair mechanisms. This vulnerability provides the biological rationale for synthetic lethality with poly(ADP-ribose) polymerase (PARP) inhibition. Accordingly, PARP inhibitors such as olaparib and talazoparib have shown clinical benefit in appropriately selected patients with HER2-negative breast cancer and germline BRCA1/2 pathogenic variants [91,92]. However, HRD is not equivalent to a BRCA1/2 mutation, since homologous recombination can be compromised by other genetic or molecular mechanisms.

Other mutational processes identified in breast cancer include APOBEC-associated mutagenesis, mismatch-repair deficiency-associated mutagenesis and POLE-associated hypermutation [93]. These signatures provide information on mechanisms of genomic instability, but differ considerably in frequency and clinical applicability. Mismatch-repair deficiency and POLE-driven hypermutation are uncommon in breast cancer, whereas APOBEC-associated signatures are relatively common [93,94]. The dMMR/MSI-high tumors are able to accumulate a large number of mutations and may generate increased neoantigen formation providing a biological rationale for immune-checkpoint inhibition. But this therapeutic principle is valid only for biomarker-positive tumors, which have been properly confirmed, and should not be generalized to breast cancer as a whole [95]. The therapeutic relevance of molecular biomarkers also depends on the tumor-immune microenvironment. Differences in immune-cell infiltration, stromal composition and tumor heterogeneity may influence the relationship between molecular alterations and response to immune-based therapies, and therefore biomarker interpretation should consider both tumor-intrinsic alterations and the surrounding immune context rather than a single molecular feature [96].

Thus, the clinical utility of mutational profiling is contingent on the specific alteration or mutational process identified and the strength of the evidence supporting its clinical application. Combining mutational information with transcriptomic, epigenomic and proteomic data may further characterize tumor biology and identify clinically relevant molecular patterns, although standardization of the analyses and prospective validation are needed before emerging signatures can be routinely incorporated into treatment decisions [97].

### 3.3. MicroRNA Signatures

MicroRNAs (miRNAs) are small non-coding RNAs that regulate gene expression primarily by sequence-dependent interaction with target messenger RNAs (mRNAs) resulting in translational repression and/or mRNA degradation [98]. Changes in miRNA expression can affect breast cancer proliferation, apoptosis, epithelial–mesenchymal transition (EMT), metastasis, and treatment resistance [68]. For example, miR-21 is often overexpressed and may suppress tumor-suppressive pathways such as PTEN-related signaling, leading to enhanced PI3K-AKT activity and cell survival [99]. In contrast, the miR-200 family acts by inhibiting transcription factors associated with EMT such as ZEB1 and ZEB2 and so may contribute to the maintenance of an epithelial phenotype [100].

Expression profiles of miRNAs have been examined for classifying breast cancer and predicting response to treatment. miRNA profiles were reported to be different in luminal, HER2-associated and basal-like tumors. miR-221/222 and miR-155 were associated with endocrine and chemotherapy resistance, respectively [101]. However, such associations should not be interpreted as evidence for clinically validated diagnostic or predictive biomarkers, as miRNA profiles vary depending on tumor subtype, cellular composition, treatment exposure and the biological compartment analyzed.

Circulating miRNAs are attractive liquid-biopsy biomarkers due to their relative stability in blood through association with proteins, lipoproteins or extracellular vesicles [102]. They therefore have been studied for non-invasive diagnosis, prognosis and treatment monitoring. However, differences in blood collection, hemolysis, RNA isolation, normalization and measurement platforms may significantly affect reported miRNA levels and contribute to inconsistent signatures between studies [102]. Therapeutic approaches using anti-miRs or miRNA mimics are also under investigation but delivery, tissue specificity, off-target effects and the broad spectrum of miRNA targets still represent important challenges [103].

## 4. Advanced Technologies for Biomarker Detection

The detection of tumor-associated biomarkers in tissue and liquid biopsies depends on analytical technologies with different molecular targets, sensitivity, throughput, and clinical applications [103]. Immunohistochemistry (IHC) and fluorescence in situ hybridization (FISH) continue to play an important role in the evaluation of breast cancer for assessment of protein expression and gene amplification [104]. However, the development of next-generation sequencing (NGS), digital PCR and proteomics has expanded the scope of biomarker analysis to allow detection of low-frequency genomic alterations, characterization of circulating tumor cells (CTCs) and measurement of protein and other molecular profiles [18]. These approaches are particularly pertinent to liquid biopsy, where tumor-derived material may exist at low levels and sensitive analytical methods may be necessary for reliable detection [42].

**Figure 2:**
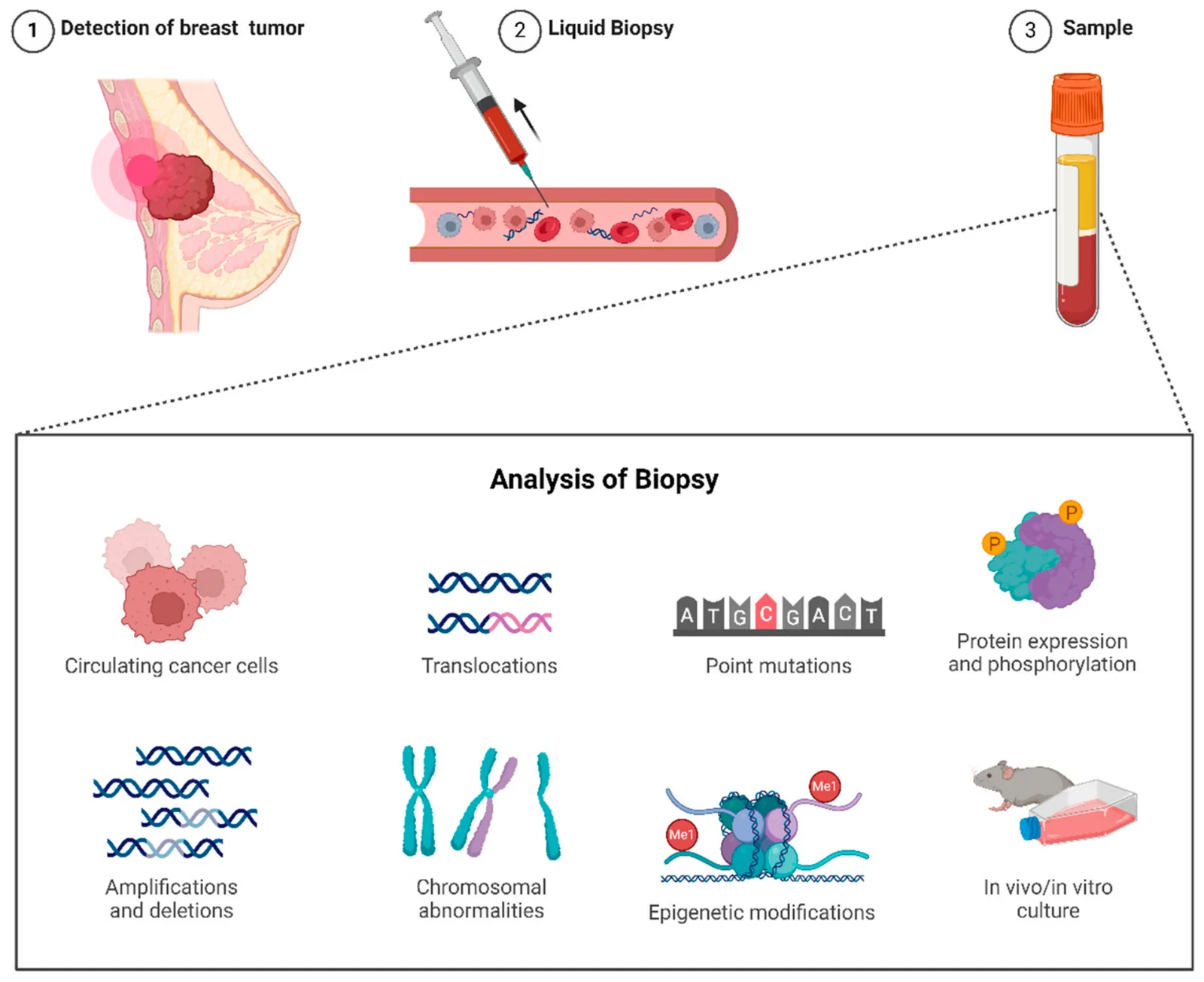
Liquid biopsy process and biomarker analysis in breast cancer.

NGS allows simultaneous analysis of multiple genomic regions and can detect mutations, copy-number alterations and other molecular aberrations. Digital PCR allows highly sensitive and quantitative detection of predefined nucleic-acid targets [105]. Proteomic approaches complement the genomic analyses by measuring proteins and signaling related changes that may not be directly inferred from alterations in DNA. Together, these technologies may enhance molecular characterization and facilitate longitudinal assessment of tumor-associated biomarkers [18]. However, analytical sensitivity does not translate to clinical utility. Assay performance depends on sample quality, target abundance, assay design and appropriate analytical thresholds. The principles, advantages and limitations of NGS, digital PCR and proteomic approaches are discussed below [106] Figure 2.

### 4.1. Next-Generation Sequencing

Next-generation sequencing (NGS) enables massively parallel, high-throughput sequencing of DNA and RNA and has greatly expanded the characterization of tumor-associated genomic alterations, depending on the assay [107]. NGS can detect single-nucleotide variants, insertions and deletions, copy number alterations, gene fusions, and other molecular features. NGS sequences millions of DNA molecules in parallel, unlike Sanger sequencing that generally sequences single DNA fragments at a time and can be used for tissue and liquid-biopsy analysis [108]. NGS workflows typically include nucleic-acid extraction, fragmentation or target enrichment, adapter ligation, library preparation, sequencing and bioinformatic analysis. Amplification may be done by bridge PCR or emulsion PCR depending on the platform, and sequencing may be done based on different signal-detection chemistries. The resulting reads are mapped to a reference genome and analyzed to identify sequence variants and other genomic alterations [109].

Targeted NGS panels are commonly used in breast cancer to screen clinically relevant genes including BRCA1, BRCA2, TP53, PIK3CA and ESR1 [110]. Detection of specific alterations can guide treatment choice in well-defined clinical scenarios, such as the identification of PIK3CA alterations relevant to PI3K-pathway inhibition or BRCA1/2 pathogenic variants relevant to PARP-inhibitor eligibility. However, the clinical significance of a genomic alteration is dependent on the variant, assay characteristics, and patient population and not detection alone [111]. WES/WGS are also used for research purposes to characterize less common alterations and mutational signatures and to investigate mechanisms that are associated with treatment response or resistance [112].

One important application of NGS in liquid biopsy is ultra-deep sequencing of circulating tumor DNA (ctDNA) [113]. Conventional sequencing may have insufficient sensitivity for detection of low-frequency variants, as ctDNA may constitute only a small proportion of circulating cell-free DNA. Error-corrected sequencing, unique molecular identifiers and targeted enrichment approaches can reduce technical errors and improve detection of rare variants [114]. These approaches are being investigated for molecular residual disease assessment, longitudinal monitoring, and detection of emergent resistance-associated alterations, but analytical detection alone does not provide clinical utility or demonstrate improved patient outcomes.

However, NGS is still limited by assay cost, turnaround time, preanalytical requirements and need for specialized bioinformatic analysis [115]. For liquid biopsy applications, sequencing errors and background variants can confound the interpretation of very low-frequency signals, necessitating stringent quality control and validated variant-calling pipelines. Thus, current research aims to improve error correction, sequencing efficiency, analytical sensitivity, and standardization to improve the clinical utility of NGS-based biomarker detection [116].

### 4.2. Digital PCR

Digital polymerase chain reaction (dPCR) is an advanced form of PCR that enables highly sensitive detection and absolute quantification of low-abundance nucleic-acid targets [117]. Unlike quantitative PCR (qPCR) that measures the fluorescence generated during amplification in a bulk reaction, dPCR partitions the sample into thousands to millions of separate reactions, each with zero, one, or a few target molecules. This partitioning facilitates the detection of rare variants and allows absolute target quantification without the need of an external standard curve [117,118].

The fundamental dPCR workflow is sample partitioning, endpoint PCR amplification, and fluorescence-based classification of individual partitions [119]. The DNA is randomly distributed into droplets or microfluidic wells, which are then amplified separately. Partitions are termed positive or negative depending upon whether or not a fluorescent signal is present. The absolute concentration of the target sequence is then estimated based on the proportion of positive and negative partitions using Poisson statistical analysis [120]. Thus, dPCR can offer high analytical sensitivity and reproducibility, especially when the target is present at low allele fraction [121].

In breast cancer, digital PCR has been employed for the detection and quantification of low-frequency alterations in circulating tumor DNA (ctDNA) including PIK3CA and ESR1 mutations in hormone receptor-positive disease. Serial analysis of these alterations may assist in characterization of emerging molecular changes during endocrine therapy and in the investigation of mechanisms of acquired resistance [122]. However, the detection of a resistance-associated mutation does not prove that treatment modification based on dPCR leads to improved clinical outcomes. Methylation-based biomarkers have also been investigated by dPCR. Aberrant methylation of tumor-associated genes, including BRCA1 and RASSF1A, can be detected in circulating DNA and may have potential for diagnosis or disease monitoring [123,124]. However, the clinical utility of such methylation assays needs analytical validation and prospective evaluation in appropriately defined patient populations.

A major advantage of dPCR is the sensitive quantification of pre-defined targets combined with relatively rapid analysis and simpler data interpretation than broad sequencing approaches. Its major limitation is that usually prior knowledge of the target sequence is required, and thus it has limited power to discover unexpected or novel alterations [125]. Conversely, NGS can simultaneously interrogate multiple genes and genomic regions, providing a broader molecular profile. Therefore, dPCR and NGS should be regarded as complementary technologies: dPCR is highly applicable in sensitive and targeted quantification, while NGS is more suitable for wider genomic characterization [126].

### 4.3. Proteomics Approaches

Proteomics provides complementary information that complements the use of DNA- and RNA-based biomarkers that are the main tools in the diagnosis of breast cancer by characterizing proteins, extracellular-vesicle cargo and post-translational modifications (PTMs) [127]. As proteins are the ultimate functional end-products of cellular processes, proteomic profiles can provide insight into dynamic changes in signaling pathways, protein abundance, protein–protein interactions, and enzymatic activity that may not be apparent from genomic measurements alone [128].

MS-based proteomics is an important platform for proteomic biomarker discovery as it allows for the identification and relative or absolute quantification of many proteins in biological samples [129]. Typically, the workflow includes protein extraction, enzymatic digestion, typically using trypsin, chromatographic separation and mass spectrometric (MS) analysis of ionized peptides based on their mass to charge (*m*/*z*) ratios. Peptide identification, quantitative reproducibility and proteome coverage have been improved by tandem mass spectrometry (MS/MS), high-resolution instruments and data-independent acquisition (DIA) [129].

In breast cancer, circulating proteins such as CA15-3, carcinoembryonic antigen (CEA) and the HER2 extracellular domain have been investigated mostly for disease monitoring, but their clinical performance and specificity vary and they are not established as stand-alone screening biomarkers [130]. Proteomic analysis of extracellular vesicles has also identified tumor-associated proteins involved in cell signaling, immune regulation, and metastatic processes, including TGF-β, HSP70 and integrins [131]. These findings may provide mechanistic understanding of tumor–microenvironment interactions, but candidate proteomic signatures need analytical and clinical validation prior to routine diagnostic use.

Emerging approaches such as phosphoproteomics and single-cell proteomics can further characterize signaling activity and cellular heterogeneity, respectively, thereby opening opportunities for biomarker discovery and identification of potentially targetable pathways [132]. Proteomic measurements are influenced by sample preparation, protein degradation, dynamic concentration ranges, and platform-specific analytical procedures [128]. Thus, a differential protein pattern alone does not prove clinical utility, and prospective validation is required to determine if a proteomic biomarker improves diagnosis, prognosis, treatment selection or disease monitoring.

## 5. Clinical Applications of Liquid Biopsies and Molecular Signatures

Increased opportunities for molecular profiling, risk assessment, treatment selection, and longitudinal disease monitoring have been provided by the integration of liquid biopsy and molecular characterization in breast cancer management [22]. A single tissue specimen only represents a specific anatomical site and time point; however, traditional histopathology and tissue biopsy are still essential as they provide direct information on tumor morphology and tissue biomarkers [133]. Instead, liquid biopsy can provide serial measurements of circulating tumor-associated material such as circulating tumor cells (CTCs), circulating tumor DNA (ctDNA), and extracellular vesicles (EVs) [22]. However, the clinical utility of these approaches depends on the biomarker, assay performance and level of clinical evidence. In particular, analytical detection of a molecular alteration should be distinguished from proven prognostic value, utility in treatment selection, or improved outcomes in prospective interventional studies [134]. Current applications include molecular characterization and monitoring of treatment, while several applications, including screening and minimal residual disease (MRD)-guided treatment, are under prospective evaluation [123].

### 5.1. Early Detection and Screening

Early detection is one of the most actively investigated applications of liquid biopsy in breast cancer. Tumor-derived DNA, RNA, proteins and extracellular vesicles can reach the circulation via processes like apoptosis, necrosis and active cellular secretion, producing measurable molecular signals that may occur before or concurrent with clinically detectable disease [135]. Among the approaches being considered are detection of tumor-associated mutations, DNA-methylation changes, circulating RNAs, and EV-associated molecular cargo [136]. ctDNA can carry alterations in genes such as TP53 and PIK3CA, but the amount of tumor-derived DNA in early-stage disease may be very low, limiting analytical sensitivity and making a negative result not sufficient to exclude cancer [8].

Epigenetic changes may occur during breast tumorigenesis; therefore, DNA-methylation biomarkers are also studied. Aberrant methylation of genes like RASSF1A and BRCA1 has been reported in breast cancer and may provide candidate signals for detection in blood [137]. Similarly, proteins and RNA species such as microRNAs, which are present in EVs and exosomes, have been studied as potential early-detection biomarkers [138]. Circulating or exosomal miRNAs such as miR-21 and miR-155 have been reported to be altered in patients with breast cancer. However, these should be treated as candidate biomarkers rather than established screening tests, as reported signatures vary across populations, sample types, analytical methods and disease stages [139].

Despite promising diagnostic performance reported in many proof-of-concept studies, liquid biopsy has not yet succeeded in replacing established breast cancer screening modalities. Major hurdles include the low abundance of tumor-derived material in early disease, biological heterogeneity, false-positive and false-negative results, and the need for standardized analytical methods [140]. The lower sensitivity and positive predictive value may be related to low tumor shedding, and overdiagnosis may be due to false-positive results and the detection of biologically indolent abnormalities. Thus, the analytical detection of circulating biomarkers should not be confused with successful population screening, and candidate molecular pathways need to be mechanistically and clinically validated [141]. Multimarker approaches combining complementary signals such as ctDNA mutations, DNA methylation patterns and circulating or EV-associated RNA could improve discrimination, but their clinical value needs validation in large prospective screening populations, and proof of their use improving clinically relevant outcomes [140].

### 5.2. Prognosis and Treatment Selection

Molecular characterization has improved conventional clinicopathological risk factors such as tumor size, lymph-node status, histological features and hormone-receptor and HER2 status, improving risk assessment and treatment choice in breast cancer [142]. Gene-expression assays may provide prognostic information on the risk of recurrence, but their predictive value is assay- and population-specific and should not be extrapolated from their prognostic performance [143]. Oncotype DX, MammaPrint and Prosigna use different gene sets and analytical approaches and thus answer related but different clinical questions. Oncotype DX measures 21 gene expression to produce a Recurrence Score that provides prognostic information and can guide decisions regarding adjuvant chemotherapy in defined populations [143]. Evidence from prospective studies such as TAILORx supports treatment decision-making in selected patients with hormone receptor-positive, HER2-negative early breast cancer, but chemotherapy benefit is contingent upon clinical and genomic risk and patient characteristics, so fixed statements that all patients with a score ≤ 25 should avoid chemotherapy or that all patients with a score > 25 benefit from chemotherapy are inappropriate [143].

Liquid-biopsy biomarkers are a complementary approach to characterize molecular evolution, particularly in advanced disease. Acquired ESR1 mutations may develop during endocrine therapy in ER-positive/HER2-negative breast cancer, and are associated with resistance to aromatase inhibitors [144]. Thus, detection of ESR1 mutations in ctDNA can provide clinically relevant information for treatment selection in appropriately defined patients. Prospective data, such as the EMERALD trial, support the use of ESR1 mutation status to select patients for the oral selective estrogen-receptor degrader (SERD) elacestrant in advanced or metastatic disease, rather than just considering ESR1 detection as a marker of resistance [144,145]. Importantly, the detection of an ESR1 mutation alone does not necessarily mean that all ctDNA-guided treatment switches improve outcomes and the clinical context, prior therapy, and available treatment options need to be taken into account [144].

Another example of biomarker-driven treatment choice is PIK3CA alterations. PIK3CA mutations can be activated in hormone receptor-positive, HER2-negative breast cancer, and activate the PI3K-AKT signaling pathway. Data from the SOLAR-1 trial support the use of alpelisib in combination with fulvestrant for appropriately selected patients with advanced PIK3CA-mutated disease [89]. Molecular testing may be performed on tissue or plasma ctDNA in this setting, with the caveat that plasma testing may be less sensitive, especially in the setting of low tumor shedding, and tissue testing should be considered when plasma testing is negative [111]. These examples illustrate the difference between prognostic biomarkers that inform about disease outcome and predictive biomarkers that identify patients more likely to benefit from a particular treatment. The wider use of serial ctDNA to guide treatment changes is an area of active clinical investigation and will require prospective interventional evidence to demonstrate that biomarker-guided management improves patient outcomes [134].

### 5.3. Monitoring Treatment Response and Disease Progression

Liquid biopsy is an adjunct to conventional imaging for longitudinal assessment of breast cancer, as molecular changes may occur before radiographic progression. Therefore, serial analysis of circulating tumor DNA (ctDNA) can provide information on changes in tumor-derived molecular signals during therapy [33]. A decrease in ctDNA after treatment initiation may correlate with response, whereas stable or increasing ctDNA may suggest residual molecular disease or the development of resistance. Importantly, ctDNA kinetics should be used as a biomarker of molecular response, not as a direct surrogate for radiographic response or clinical benefit. Discordance between molecular and radiographic responses may arise because ctDNA reflects tumor shedding and molecular evolution rather than simply tumor volume [146]. Such discordance should therefore be considered in the light of imaging, clinical findings and treatment, and not to prompt treatment change based on ctDNA alone. Persistent ctDNA following systemic therapy completion has been associated with increased risk of subsequent recurrence in breast cancer, supporting its prognostic value [146].

Circulating tumor cells (CTCs) offer another strategy for longitudinal monitoring, especially in the case of metastatic breast cancer. CTC enumeration has been established in a prognostic manner, with higher CTC counts generally associated with worse clinical outcomes [147]. Individual CTCs can be molecularly characterized to further reveal phenotypic and genomic heterogeneity that may be distinct from the primary tumor. For example, changes in HER2 expression have been investigated as an indicator of tumor progression, and may provide information relevant to treatment selection [148]. However, CTC measurements are dependent on the detection platform and biologic differences in tumor-cell shedding, and changes in CTC counts have not consistently been shown to improve outcomes when used alone to guide treatment.

One important emerging application is the detection of molecular minimal residual disease (MRD) following treatment with curative intent. Very sensitive ctDNA assays, often tailored for a specific patient, can detect tumor-derived DNA when conventional imaging does not show evidence of disease [149]. ctDNA positivity after surgery or systemic therapy is associated with a substantially increased risk of recurrence and therefore carries strong prognostic significance [150]. However, prognostic MRD detection should be distinguished from the evidence that changing treatment on the basis of ctDNA alone improves survival or other patient outcomes. Therefore, prospective interventional trials are now testing whether ctDNA-based escalation or de-escalation of therapy can turn molecular detection into clinical benefit [134].

Serial ctDNA measurements should be interpreted considering also biological and technical variability. Factors influencing ctDNA levels include tumor burden, anatomical site, tumor-cell shedding, treatment effects, DNA clearance and the variant of interest [151]. Sample processing, assay design, sequencing depth and reporting thresholds also can further influence measurements across platforms. Thus, standard analytical procedures are needed for meaningful longitudinal comparison [152]. Machine learning approaches could be useful to integrate serial ctDNA, CTC, imaging and clinical variables but such models require independent validation before routine clinical implementation [153]. Overall, liquid biopsy has shown prognostic and monitoring applications but broader use for treatment adaptation remains an active area of prospective investigation.

## 6. Artificial Intelligence and Machine Learning in Breast Cancer Diagnostics

The increasing amount of imaging, genomic, transcriptomic, proteomic and liquid-biopsy data in breast cancer diagnostics makes the application of artificial intelligence (AI) and machine learning (ML) for data analysis and biomarker interpretation more and more promising [154]. The backbone of conventional diagnostic workflows is the expert interpretation of histopathology, imaging and molecular measurements, which can be influenced by inter-observer variability and challenges in integrating heterogeneous data sets [155]. AI and ML methods can handle high-dimensional data, identify intricate patterns, and build classification or prediction models that could supplement clinical expertise. Deep-learning and neural-network methods have been explored in breast cancer for mammographic and radiomic analysis, digital pathology, molecular profiling, and multimodal data integration [156].

AI-based approaches have also been explored for biomarker interpretation and longitudinal analysis of liquid-biopsies. Models applied to ctDNA may detect patterns in the variant profile, fragment profile, or other molecular features that are difficult to interpret through individual biomarkers alone [157]. Similarly, integrating CTC, extracellular-vesicle and molecular data with clinical or imaging variables may provide a more comprehensive picture of tumor evolution [16]. However, better performance in a development or retrospective dataset should not be considered as proof of clinical utility. Dataset composition, class imbalance, batch effects, missing data, and differences between training and clinical populations might affect model performance. The external validation, prospective assessment, interpretability and adequate regulatory oversight are still critical before AI models can be included in routine diagnostic decision-making.

### 6.1. AI-Assisted Biomarker Discovery

The discovery of genomic, transcriptomic, proteomic or other molecular biomarkers involves the analysis of high dimensional data sets, where relevant signals may include complex interactions among many variables. AI and ML methods can support this process in molecular pattern finding, dimension reduction and feature selection related to disease phenotypes or clinical outcomes [158]. Unsupervised techniques such as hierarchical clustering and principal component analysis (PCA) can identify groups of patients that are similar at the level of the molecules and reveal patterns that complement conventional clinicopathological classification [159]. Then supervised learning techniques can be applied to evaluate whether selected molecular features associate with diagnostic categories, risk of recurrence, treatment response, or other pre-specified endpoints [159].

Deep-learning methods, such as convolutional neural networks (CNNs) and other neural network architectures, have been increasingly investigated for multi-omics integration and are capable of analyzing complex molecular and imaging datasets [158]. These integrative approaches that integrate data from genomic alterations, gene expression, and protein measurements, can reveal interactions that are difficult to find through single-omic analyses [158]. Network-based approaches have also been used to investigate regulatory interactions of long non-coding RNAs (lncRNAs), microRNAs and protein-coding genes, resulting in potential molecular signatures for further experimental and clinical validation [160].

AI is also being used for biomarker analysis in liquid biopsies. Computerized models can analyze ctDNA mutation patterns, fragmentomic features, methylation patterns and combinations of circulating biomarkers to improve the classification of molecular states and detect trends associated with disease recurrence or treatment response [161]. Many reported models remain at the biomarker-discovery or retrospective-validation stage. In particular, low ctDNA abundance in early-stage disease, biological heterogeneity, assay-specific measurement differences, and limited external validation may restrict generalizability [161].

Therefore, the translation of AI-derived biomarkers to clinical practice requires more than high predictive performance in a discovery cohort. Independent external validation, standardized data processing pipelines, transparent reporting of model development, assessment of calibration and clinical utility, and prospective evaluation are needed for reproducibility and clinical relevance [161]. These requirements are particularly important when AI models include multi-omics or serial liquid biopsy data, as differences in sample handling, measurement platforms, normalization and missing data patterns can lead to systematic bias. Hence, AI should be viewed as a tool for the discovery and interpretation of biomarkers, not as a replacement for biological validation or clinical evidence [158,161].

### 6.2. Machine Learning Models for Data Interpretation

Heterogenous breast cancer datasets including medical imaging, digital pathology, genomic profiles, liquid-biopsy measurements and clinical data have been increasingly explored for interpretation by machine learning (ML) models [162]. ML methods can be broadly divided into supervised, unsupervised, and reinforcement learning, but their applications and clinical validation are substantially different [163]. Notably, high classification or prediction accuracy in a retrospective dataset does not inherently guarantee clinical utility, and model performance needs to be assessed using independent datasets, proper calibration, and clinically relevant endpoints.

#### 6.2.1. Supervised Learning for Breast Cancer Detection

Supervised learning uses labeled datasets, where the outcome of interest has already been defined, and algorithms such as support vector machines (SVMs), decision tree models, random forests, and neural networks learn relationships between input features and diagnostic or clinical outcomes [164]. Deep-learning convolutional neural networks (CNNs) have been used in breast imaging including mammography, ultrasound, and MRI to detect and classify breast lesions and to extract imaging features that may be difficult to quantify consistently by visual assessment [165]. However, claims of near-perfect diagnostic accuracy are generally not appropriate for clinical practice as the reported performance varies with dataset composition, imaging modality, disease prevalence, and external validation [166].

Imaging findings can be combined with clinical features, family history or molecular information in multimodal models. In digital pathology, deep-learning models have been applied to whole-slide images to detect tumors, grade them, assess proliferation, and predict molecular characteristics [167]. More recent architectures such as vision transformers (ViTs) have expanded image representation beyond traditional convolutional approaches and generative models have been explored for applications such as image reconstruction and virtual staining [168]. These methods may improve reproducibility and reduce some types of observer variability, but they still depend on image quality, representative training data, appropriate annotation, and validation between institutions.

#### 6.2.2. Unsupervised Learning for Tumor Subtyping and Patient Stratification

Unsupervised learning identifies patterns without requiring predefined outcome labels, thus being useful for exploring biological heterogeneity. Clustering methods (e.g., k-means, hierarchical clustering) and dimensionality-reduction approaches (e.g., principal component analysis (PCA), t-SNE, UMAP) are capable of analyzing high-dimensional genomic, transcriptomic, epigenetic or imaging datasets [169]. These approaches have led to the identification of molecularly distinct patient groups and patterns of immune infiltration, metabolic activity, stromal composition and other biological characteristics in breast cancer research [170].

However, a cluster identified algorithmically does not automatically represent a clinically meaningful subtype. New molecular groups need biological characterization, reproducibility in independent cohorts, and validation of their association with clinically relevant outcomes [171]. Similarly, an association with survival does not imply that placing patients in a particular cluster improves treatment selection. Thus, ML-derived classifications should be considered candidate biological or prognostic frameworks until validated independently.

#### 6.2.3. AI-Driven Predictive Models for Therapy Response

ML models are also under development to predict treatment response and clinical outcomes based on a combination of imaging, molecular and clinical variables. Models may include gene-expression signatures, proliferation markers, genomic alterations, ctDNA measurements and clinical features to predict risk of recurrence or treatment response [172]. Such approaches reveal complex relationships among multiple variables that may not be captured by conventional statistical models. However, if established biomarkers such as Oncotype DX are incorporated into an ML model, the model should not be interpreted as independently establishing the predictive value of the underlying assay or demonstrating chemotherapy benefit. Prognostic prediction and prediction of treatment benefit are still different clinical questions [143].

Reinforcement learning (RL) differs from traditional supervised learning in that an algorithm learns via sequential interactions with an environment and assesses alternative actions against predefined outcomes or reward functions. In oncology research, RL has been explored for adaptive treatment strategies with longitudinal clinical data [173]. But most of those applications remain under investigation. Retrospective treatment data can be subject to confounding by indication and other biases such that a treatment strategy derived from a model cannot be assumed to improve patient outcomes in the absence of prospective evaluation [174].

Therefore, there are several challenges that limit the immediate clinical deployment of AI-based breast cancer models. Explainability and interpretability are critical as complex deep-learning systems may offer predictions but do not easily reveal the biological or clinical rationale behind them [175]. Explainable AI (XAI) methods can provide information about feature importance, image regions, model decisions or prediction uncertainty, but these methods do not necessarily establish causal relationships. Another important issue is data heterogeneity: different scanners, pathology workflows, sequencing platforms, patient populations, and clinical practices may reduce the generalizability of the model. Models developed in single-center or geographically limited datasets may perform differently in external populations, particularly when demographic and biological diversity is limited [154,175].

Thus, robust clinical translation needs external and preferably multicenter validation, transparent reporting, appropriate management of missing data and class imbalance, assessment of calibration and discrimination, and evaluation of clinically meaningful outcomes [154]. For liquid-biopsy and multi-omics applications, further challenges include batch effect, variable assay sensitivity and the high dimensionality of molecular features. Future ML systems may include imaging, pathology, genomics, proteomics, and serial liquid-biopsy measurements, but their clinical role should be defined by rigorous validation rather than extrapolated from computational performance alone [175].

## 7. Commercially Available Diagnostic Assays

Molecular diagnostics have evolved to provide information on tumor biology and recurrence risk that complement conventional histopathology and receptor assessment, expanding the management of breast cancer. Multigene expression assays may refine risk stratification and in selected patient populations guide adjuvant treatment decisions [143]. However, these assays address different clinical questions and are not interchangeable. In particular, prognostic information about risk of recurrence should be distinguished from evidence that a biomarker predicts benefit from treatment [143]. Liquid-biopsy assays leveraging circulating tumor DNA (ctDNA) and circulating tumor cells (CTCs) provide opportunities for serial molecular assessment including characterization of tumor evolution and treatment resistance; however, analytical detection alone is not sufficient to establish clinical utility or demonstrate improved outcomes when used to guide treatment [176]. This implies that commercial applications of liquid biopsy are still less widespread than established tissue-based multigene assays.

### 7.1. Review of Current Molecular Diagnostic Tests

Among the established multigene assays in breast cancer, Oncotype DX, MammaPrint and Prosigna offer different approaches for molecular risk assessment [80,81]. Oncotype DX measures the expression of 21 genes and generates a Recurrence Score, which provides prognostic information on distant recurrence and in selected populations with hormone receptor-positive (HR+), HER2-negative early breast cancer can help guide the decision-making process regarding adjuvant chemotherapy [79]. The prospective TAILORx trial provided evidence for use of Recurrence Score results to guide chemotherapy decisions in appropriately selected node-negative patients and demonstrated that many patients with intermediate-range scores could be treated without chemotherapy while chemotherapy benefit was concentrated in specific higher risk groups and varied according to age and clinical characteristics [143]. Thus, TAILORx does not demonstrate a single Recurrence Score threshold that defines the benefit of chemotherapy in all patients, but rather informs treatment decision-making in well-defined patient populations.

MammaPrint evaluates a 70-gene expression signature and classifies tumors in genomic risk groups [141]. Oncotype DX provides a numerical Recurrence Score. MammaPrint provides a genomic risk classification that can be combined with standard clinical risk assessment [80,81]. The recent MINDACT trial assessed the clinical utility of MammaPrint in patients with concordant and discordant genomic and clinicopathological risk classifications and showed that selected patients with clinically high but genomically low risk could have chemotherapy omitted with a low rate of distant recurrence [177]. These results support the application of MammaPrint in treatment decision-making in appropriately selected populations of early breast cancer, but also show that genomic risk should be considered together with clinical characteristics and not as an independent determinant of treatment.

Prosigna utilizes the PAM50 gene-expression framework to identify intrinsic molecular subtypes and calculate a Risk of Recurrence (ROR) score [178]. The assay provides information on molecular subtype and estimated risk of recurrence and has been demonstrated to have prognostic value particularly in postmenopausal patients with HR-positive early breast cancer [179]. However, its clinical role is different from Oncotype DX and MammaPrint, and evidence of prognostic performance should not be interpreted as equivalent to prospective evidence that the assay predicts benefit from a specific systemic treatment. Thus, the selection and interpretation of a multigene assay should be based on the clinical context, patient population, intended endpoint, and the level of evidence supporting its use [143,179]. A comparison of the major commercially available assays is summarized in Table 2.

Collectively, these assays highlight an important difference between molecular risk stratification and treatment prediction. Oncotype DX and MammaPrint have prospective evidence to support treatment decision making in specific clinical populations, whereas Prosigna has been primarily developed as a prognostic assay. Thus, none of these tests should be regarded as absolute in determining whether chemotherapy is needed or not.

### 7.2. Emerging Liquid Biopsy-Based Tests

Assays based on liquid biopsies are increasingly studied and, for certain applications, used clinically to characterize tumor-derived molecular alterations without the need for a new tissue biopsy [176]. These assays analyze circulating tumor DNA (ctDNA), circulating tumor cells (CTCs), or extracellular vesicle-associated material and can offer serial molecular measurements over the course of disease [176]. However, their clinical roles differ depending on the assay and intended application. Some plasma-based assays are used for genomic profiling and treatment selection in advanced disease, while tumor-informed ctDNA assays are mainly being evaluated for molecular residual disease (MRD) and recurrence-risk assessment [176]. Thus, detection of a circulating biomarker needs to be differentiated from proof that biomarker-guided treatment modification improves clinical outcomes. It is important to distinguish the regulatory status and intended use of an assay from its clinical adoption and from putative evidence of improved patient outcomes through its use.

Guardant360 CDx is a next-generation sequencing assay based on plasma, designed to detect genomic alterations in circulating cell-free DNA (cfDNA) and to identify molecular alterations that may have therapeutic relevance [183]. In breast cancer, plasma genotyping can identify clinically relevant alterations, such as PIK3CA mutations and, in the appropriate clinical contexts, ESR1 mutations associated with endocrine resistance [111]. Thus, if ctDNA exists at a high enough concentration, plasma testing can be a minimally invasive method to identify an actionable alteration [176]. However, a negative plasma result does not necessarily exclude tumor alteration, as ctDNA shedding is variable between patients and disease sites. Therefore, tissue testing may remain appropriate when plasma testing is uninformative or when clinically indicated [111]. Similarly, the identification of a resistance-associated alteration provides molecular evidence of tumor evolution, but does not in and of itself demonstrate that every biomarker-guided change in therapy improves outcomes.

Signatera is a tumor-informed ctDNA assay designed for MRD detection and longitudinal recurrence-risk assessment, offering an alternative approach to liquid biopsy [184]. The assay involves sequencing a patient’s tumor tissue to identify multiple patient-specific somatic variants and then creating a personalized plasma assay to detect those variants [184]. This tumor-informed approach is distinct from fixed-panel plasma genotyping assays that interrogate a pre-defined set of genomic regions. Detection of ctDNA after therapy has been associated with a significantly increased risk of subsequent recurrence in early-stage breast cancer and in some patients it may precede a clinically detectable relapse [185]. These findings support the prognostic value of ctDNA-defined MRD. However, MRD positivity should not be considered as an indication for immediate escalation of treatment; whether a modification of adjuvant treatment based only on the detection of ctDNA improves recurrence-free or overall survival needs to be confirmed with prospective interventional evidence [176].

Therefore, the growing clinical use and investigation of liquid biopsy assays reflect their potential to complement, rather than universally replace, tissue-based assessment [39]. Serial ctDNA measurements can give insight into clonal evolution and emerging resistance that a single tissue specimen cannot, whereas CTC analysis may provide complementary information on circulating tumor-cell burden and phenotype [39]. However, assay sensitivity is affected by tumor burden, anatomical site, ctDNA shedding, sample quality, assay design and analytical thresholds. Technical standardization and reproducibility still remain important challenges, especially when measuring very-low-frequency variants or MRD [8]. Advances in error-corrected sequencing, digital PCR and computational approaches may improve analytical performance but improved sensitivity alone does not establish clinical utility.

Therefore, Guardant360 CDx and Signatera are not interchangeable liquid biopsy tests. Guardant360 CDx is a comprehensive plasma-based genomic profiling and potentially actionable alterations identification platform [180,181,183,184]. Signatera is a personalized tumor-informed assay for MRD detection and molecular recurrence risk assessment. Therefore, they have different clinical evidence, target populations, biomarkers and endpoints [184]. The integration of liquid-biopsy results with tissue profiling, imaging, clinicopathological variables and other molecular data could further improve the characterization of tumor evolution, although the clinical value of such integrated approaches requires prospective validation. These differences are summarized in Table 2.

## 8. Challenges and Limitations

Technical, biological, analytical and clinical barriers limit the potential of liquid biopsies and molecular signatures to complement conventional breast cancer diagnostics and limit their broader implementation [186]. Liquid biopsy offers the opportunity of accessing tumor-associated material without the need for repeated tissue sampling; however the concentration and composition of circulating biomarkers vary considerably based on tumor burden, anatomical site, vascularization, tumor biology and exposure to treatment [15]. Therefore, a negative liquid biopsy cannot rule out the presence of a molecular alteration or residual disease. Other challenges include preanalytical variation, assay sensitivity, platform dependent results, bioinformatic interpretation, tumor heterogeneity, cost and limited prospective evidence for some emerging clinical applications [186]. Analytical validity and reproducible detection of a biomarker are to be distinguished from its clinical validity, its prognostic value and evidence that treatment guided by it improves patient outcome [176].

### 8.1. Technical Challenges in Biomarker Detection

Reliable detection of tumor-derived material at extremely low abundance is one of the major technical challenges for liquid biopsy. This is especially relevant for ctDNA in early-stage breast cancer where the tumor-derived fraction of circulating cell-free DNA can be very small [8]. Digital PCR, targeted deep sequencing, error-corrected NGS and unique molecular identifiers can improve analytical sensitivity, but the detection of low-frequency variants is still subject to sequencing errors, background biological variation and inappropriate variant-calling thresholds [8]. Other sources of potentially misleading variant calls include clonal hematopoiesis, which can contribute somatic variants to plasma DNA that are not from the breast tumor. Thus, the increased analytical sensitivity must be accompanied by appropriate controls and interpretation of the biological origin of the variants detected [8].

CTC analysis poses a different set of technical challenges. CTCs are rare biologically heterogeneous cells that may vary in size, phenotype, epithelial-marker expression and viability [67,68,69]. Epithelial-to-mesenchymal transition or decreased expression of targeted epithelial markers will result in cells that cannot be detected by capture methods that target epithelial markers [187]. Immunomagnetic enrichment, microfluidic separation, size-based approaches, and other technologies can improve CTC recovery, but differences in enrichment and detection strategies may result in very different cell counts and molecular profiles. Thus, CTC numbers should be interpreted according to the analytical platform used and should not be assumed to be directly interchangeable with numbers generated by other technologies [187].

The analysis of extracellular vesicles (EVs) and exosomes adds another layer of preanalytical and analytical complexity. EV populations are heterogeneous and can originate from tumor cells as well as normal blood and stromal cells. Methods of isolation include ultracentrifugation, size-exclusion chromatography, precipitation and immunoaffinity capture, and they differ in terms of recovery, purity, scalability and susceptibility to contamination by non-vesicular proteins or other particles [57]. Therefore, differences in isolation protocols can affect the RNA, protein or lipid cargo being measured and lead to apparently different biomarker signatures between studies.

Main preanalytical variables are also different depending on the liquid biopsy analyte. The type of blood collection tube, the time from collection to plasma separation, temperature and storage conditions, plasma-processing procedures, and leukocyte contamination can affect the integrity of cfDNA and the measured tumor fraction for ctDNA [188]. CTC analysis is sensitive to blood volume, collection conditions, time to processing, cell viability and the enrichment or fixation procedure [189]. For EVs and exosomes, the type of anticoagulant, the processing time, centrifugation conditions, storage, hemolysis and isolation method may influence vesicle yield and cargo composition [187]. These differences imply that one preanalytical workflow cannot be used for all of the liquid-biopsy analytes, and emphasize the need for analyte-specific protocols and quality control procedures [16,24].

The biological heterogeneity further complicates the detection of biomarkers. The signals from the tumor may differ from primary to metastatic sites, and from sub-clones within the same tumor [5,73]. Treatment can impose selective pressure, altering the abundance of specific clones, thus a biomarker identified at one point in time may become undetectable and another resistant clone may emerge [5,56]. However, serial liquid-biopsy sampling may allow capture of these temporal changes, but the optimal sampling interval, clinically meaningful change in biomarker level and the relationship of molecular change to treatment outcomes remain dependent on the specific biomarker and clinical context [15,21,24,25].

### 8.2. Standardization and Reproducibility Issues

The lack of harmonization across laboratories and analytical platforms poses a major limitation to reproducibility. Variation can occur at various stages such as blood collection, sample processing, nucleic-acid extraction, library preparation, sequencing depth, PCR amplification, variant calling, and reporting [44]. For ctDNA assays, differences in sequencing depth, error correction procedures, molecular barcoding, variant allele frequency thresholds, and bioinformatic pipelines may influence the number and frequency of detected variants [116]. Thus, apparently different results may be due to methodological rather than genuine biological differences.

Standardization is also important for CTC and EV assays. The enumeration and molecular characterization of CTCs depend on the capture and detection technology used, while EV studies may differ in the isolation method, vesicle characterization, normalization strategy, and the definition of the EV population being analyzed [16]. Therefore, the direct comparison between studies might be difficult since there are no universally harmonized procedures. Reference materials, assay-specific quality controls, transparent reporting of analytical thresholds and cross-platform validation are critical to improving reproducibility [65,152,187].

Another source of methodological variation is that multigene expression assays differ in the gene sets, normalization methods, algorithms and clinical endpoints used. Therefore, Oncotype DX, MammaPrint, and Prosigna are not expected to consistently categorize risk in all patients. Differences between assays do not mean that one assay is analytically unreliable but that they measure different biological features in different populations in which each assay has been validated [75,79,80,81,177]. Therefore, genomic risk categories are not interchangeable across different assays and clinical interpretation should be based on assay-specific evidence bases.

Reproducibility becomes even more challenging when data from different molecular platforms are combined. Multi-omics datasets may have different measurement scales, distributions of features, missing observations and batch effects due to laboratory procedures or sequencing platforms [154,158,163,172]. Hence, reliable integration of datasets demands appropriate normalization, batch effect correction, quality control, feature selection and management of missing data. These computational issues are especially relevant when jointly analyzing genomic, transcriptomic, proteomic and other data types [154,158,172], and their methodological implications and possible solutions will be discussed in more detail in Section 9.1.

Tumor heterogeneity also constrains reproducibility and interpretability of molecular signatures. Intratumoral heterogeneity implies that different subclones within the same tumor can have different genomic and transcriptomic profiles [5,14,73]. Intertumoral heterogeneity leads to molecular differences between patients with seemingly similar clinicopathological characteristics. Metastatic lesions can also develop changes that are not in the primary tumor. One tissue specimen or liquid-biopsy measurement can therefore only provide a partial picture of the disease [14,15,73]. Serial liquid biopsies can complement tissue profiling by measuring temporal changes in circulating tumor-derived material but are not necessarily a complete representation of all tumor compartments. The clinical significance of serial sampling and frequency of testing are active areas of investigation [15,17,24,25,51].

### 8.3. Regulatory and Clinical Adoption Challenges

Translating molecular and liquid-biopsy assays into routine care requires evidence at multiple levels, including analytical validity, clinical validity, and clinical utility. Regulatory approval or clearance addresses specific performance and intended use requirements but should not be assumed to automatically demonstrate a test improves patient outcomes in all clinical settings [24,116,152,176]. New liquid biopsy assays may have good analytical performance but may lack prospective interventional evidence that treatment decisions based on their results improve survival, recurrence outcomes or quality of life [24,116,176]. Therefore, the regulatory status and intended use of each assay need to be taken into account together with the level of evidence.

Cost and reimbursement impact clinical adoption. NGS-based assays and other specialized molecular testing may require extensive laboratory infrastructure, sequencing capacity, bioinformatic expertise, and quality control procedures [18,97,106,116,136]. Reimbursement policies vary by healthcare system, indication and assay, and reimbursement for established genomic assays does not automatically extend to newer liquid-biopsy technologies [18,24,136]. Cost-effectiveness must be shown not just in terms of the cost of testing, but in terms of the relevance of management changes directed by the test to change treatment, avoid ineffective therapy, identify clinically actionable disease, or improve relevant patient outcomes. Therefore, assessments should be based on evidence from the intended clinical population and not assume that molecular testing is by definition cost-saving [18,24,116,176].

Appropriate interpretation of complex molecular results is also needed for clinical implementation. Large sequencing panels can identify pathogenic variants, variants of uncertain significance, and alterations whose clinical relevance is tumor type-, treatment history-, or evidence-based [97,106,116,134,152]. Thus, clinicians require access to validated interpretation frameworks and if required molecular tumor boards or specialist genomic expertise. Decision support and artificial intelligence tools may assist in the interpretation of complex data sets, but their recommendations need to be independently validated and should be used in conjunction with clinical judgment and established evidence [160].

In general, analytical sensitivity is not the sole major barrier to clinical translation. Reliable implementation requires control of preanalytical variables, reproducible laboratory procedures, appropriate computational analysis, the recognition of tumor heterogeneity, clinically validated interpretation and evidence that testing provides meaningful benefit in the intended patient population [97,106,116,134,152]. Therefore, liquid biopsy and molecular diagnostics should be considered complementary tools in precision breast cancer care, and not generic alternatives to tissue pathology or routine clinical evaluation.

## 9. Future Perspectives

More integration of molecular profiling, liquid biopsy, imaging and computational approaches will likely be seen for the future of breast cancer diagnostics. Multi-omics strategies may offer complementary information on tumor heterogeneity and molecular evolution, while serial liquid biopsy may enable longitudinal assessment of treatment-related changes. But more molecular information does not necessarily mean better clinical decisions. Future studies should therefore be directed toward the identification of reproducible biomarkers with clinically useful information beyond established clinicopathological factors and to demonstrate their value in prospective clinical settings.

### 9.1. Integrating Multi-Omics Approaches

The integration of genomics, transcriptomics, epigenomics, proteomics and metabolomics may allow a more complete characterization of breast cancer biology than single molecular platforms. However, multi-omics integration faces important methodological challenges including batch effects, measurement scale differences, normalization, missing data, high dimensionality and feature-selection instability. If not properly handled, these factors can introduce bias and overfitting, especially when a large number of molecular features are analyzed in relatively small patient cohorts. Recent multi-omics modeling studies further illustrate the potential of integrating complementary biological data layers for risk stratification while highlighting the importance of appropriate feature selection and model validation [190].

Liquid-biopsy measurements may also improve longitudinal multi-omics analysis by integrating serial ctDNA, CTC, or extracellular-vesicle data with tissue profiling, imaging, and clinical information. Such approaches may help us to understand tumor evolution and treatment resistance. However, circulating biomarkers only reflect a small fraction of the tumor ecosystem and may not be able to detect all spatially distinct tumor clones. Thus, integrated multi-omics approaches should be viewed as complementary to tissue-based assessment and the clinical utility will need cross-platform validation and prospective assessment.

Depending on the clinical objective, the integration can be a pre-defined multi-marker algorithm, a machine-learning classifier, or a sequential testing strategy where complementary biomarkers are tested at different decision points. The optimal approach would be determined by the properties of the biomarker, the information at hand, and the extent of clinical validation [191].

### 9.2. Personalized Medicine and Targeted Therapies

Molecular diagnostics have an increasingly important role in personalized treatment through biomarkers such as hormone-receptor and HER2 status, germline BRCA1/2 pathogenic variants and PIK3CA and ESR1 alterations in appropriately defined clinical settings. Serial molecular measurements to further characterize tumor evolution and emerging treatment resistance could be part of future precision oncology. However, prognostic biomarkers and biomarkers with a demonstrated value for treatment selection should be clearly differentiated.

Serial liquid biopsy can help support adaptive treatment strategies by detecting molecular changes during therapy, but detection of a molecular alteration does not in itself prove that changing therapy based on that detection improves patient outcomes. Prospective interventional studies are thus required to ascertain if liquid-biopsy-guided treatment adaptation benefits patients in the clinic. Emerging approaches such as CRISPR-based gene modification and RNA-targeted treatments may also play a role in future precision oncology, but their application in breast cancer is still investigational and further data on safety, delivery, specificity and clinical benefit are needed.

Genomic and liquid-biopsy profiling may help to identify molecular features that can potentially guide emerging gene-editing and RNA-based therapies in the future, particularly in biologically defined breast cancer subgroups such as triple-negative disease, but these approaches are still investigational and require further validation [192,193].

### 9.3. Artificial Intelligence in Precision Diagnostics

AI and ML could help to integrate and interpret ever more complex breast cancer data sets, including genomic, transcriptomic, proteomic, imaging, pathology, clinical and liquid-biopsy data. Computational methods are capable of analyzing liquid biopsy studies of ctDNA variants, methylation, fragmentomic features and longitudinal measurements. However, high performance on retrospective datasets does not tell us about clinical utility, and model generalizability can be compromised by overfitting, batch effects, missing data, class imbalance, and differences in patient populations or analytical platforms.

Therefore, future AI applications will need external and ideally multicenter validation, proper normalization and handling of missing data, robust feature selection, and assessment of calibration and clinical utility besides predictive accuracy. Explainable artificial intelligence can help to interpret complex models, but the feature importance of the model should not be taken as evidence for biological causality. In general, the clinical role of AI should be defined by rigorous validation and prospective evaluation, and not just by computational performance.

## 10. Conclusions and Clinical Implications

Liquid biopsies and molecular signatures are promising tools with increasing applicability for molecular characterization, risk assessment, treatment selection, and disease monitoring in breast cancer. ctDNA, CTCs and extracellular-vesicle biomarkers can provide longitudinal information complementary to tissue biopsy, but do not fully capture tumor heterogeneity or substitute tissue-based analysis.

Molecular assays such as Oncotype DX, MammaPrint, and Prosigna provide clinically useful risk information in selected patient populations, but their prognostic value must be distinguished from demonstrated prediction of benefit from therapy. Similarly, ctDNA can detect selected resistance-associated changes and molecular residual disease, but detection alone does not show that biomarker-guided changes in treatment improve outcomes.

Yet, despite the progress made by NGS, digital PCR, AI and multi-omics approaches, the challenges of biomarker abundance, standardization, reproducibility, tumor heterogeneity and clinical validation remain. Future progress will depend on integration of these technologies with tissue profiling and established clinical assessment, backed by strong prospective evidence. The end goal is not just more molecular sensitivity but reliable biomarkers that provide clinically actionable information and improve patient outcomes.

## Figures and Tables

**Table 1:** Comparison of liquid biopsy biomarkers in breast cancer.

Biomarker Type	Source	Advantages	Challenges	Clinical Applications	References
Circulating tumor cells (CTCs)	Blood	Provides insights into tumor aggressiveness and metastatic potential	Low abundance in circulation; requires specialized isolation techniques	Prognostic marker, therapy response monitoring, metastatic risk assessment	[67,68,69]
Circulating tumor DNA (ctDNA)	Blood (plasma)	Non-invasive, real-time tracking of tumor evolution and therapy resistance	Low levels in early-stage cancer, potential contamination with normal cfDNA	Early detection, minimal residual disease (MRD) monitoring, resistance mutation detection	[43,70]
Exosomal RNA and proteins	Blood, urine	Encapsulated within vesicles, protected from degradation	Isolation and characterization methods require standardization	Early-stage detection, immune response modulation, therapy resistance prediction	[71]
Methylated ctDNA	Blood (plasma)	Detects epigenetic alterations associated with tumorigenesis	Requires highly sensitive techniques for low-level detection	Early cancer screening, prognosis prediction, treatment response evaluation	[72]

**Table 2:** Comparison of commercially available molecular diagnostic assays in breast cancer.

Test Name	Technology	Primary Clinical Use	Key Advantages	Limitations	References
Oncotype DX	RT-qPCR gene expression analysis	Predicts recurrence risk and chemotherapy benefit in HR+/HER2- breast cancer	Identifies patients who can safely avoid chemotherapy, validated in large trials	Limited to HR+/HER2- tumors; not applicable to triple-negative or HER2+ breast cancer	[180,181]
MammaPrint	Microarray-based gene expression profiling	Stratifies early-stage breast cancer patients into high- and low-risk categories	Provides binary risk classification, reducing uncertainty in chemotherapy decisions	Requires fresh or frozen tissue, limiting sample availability	[182]
Prosigna (PAM50)	RT-qPCR-based intrinsic subtype classification	Predicts long-term recurrence risk and categorizes breast cancer subtypes	Provides intrinsic molecular subtyping (Luminal A, Luminal B, HER2-enriched, basal-like)	Limited utility in premenopausal women and patients receiving neoadjuvant therapy	[178]
Guardant360	NGS-based ctDNA analysis from blood	Identifies actionable mutations in metastatic breast cancer	Non-invasive alternative to tissue biopsy for real-time therapy selection	Sensitivity decreases in early-stage disease due to low ctDNA levels	[183]
Signatera	Personalized tumor-informed ctDNA sequencing	Detects minimal residual disease (MRD) and early relapse risk	High specificity as it tracks patient-specific mutations	Requires initial whole-exome sequencing of primary tumor for personalized assay design	[184]

## Data Availability

No new data were created or analyzed in this study. Data sharing is not applicable to this article.
